# *De novo* assembling a complete mitochondrial genome of *Pedicularis rex* (Orobanchaceae) using GetOrganelle toolkit

**DOI:** 10.1080/23802359.2020.1722038

**Published:** 2020-02-03

**Authors:** Xin Li, Chun-Yan Lin, Jun-Bo Yang, Wen-Bin Yu

**Affiliations:** a Center for Integrative Conservation, Xishuangbanna Tropical Botanical Garden, Chinese Academy of Sciences, Mengla, Yunnan, China;; b University of Chinese Academy of Sciences, Shijingshan District, Beijing, China;; c Center of Conservation Biology, Core Botanical Gardens, Chinese Academy of Sciences, Mengla, Yunnan, China;; d Plant Germplasm and Genomics Center, Germplasm Bank of Wild Species, Kunming Institute of Botany, Chinese Academy of Sciences, Kunming, Yunnan, China;; e Southeast Asia Biodiversity Research Institute, Chinese Academy of Science, Yezin, Nay Pyi Taw, Myanmar

**Keywords:** Hemiparasitic plant, mitogenome, *Pedicularis*, whole genome sequencing data

## Abstract

We reported the first mitogenome of *Pedicularis* from *P. rex* (Orobanchaceae), which is endemic to SW China. The complete mitochondrial genome (mitogenome or chondriome) was a single circular chromosome that was 219,859 bp in length. It contains 56 genes, including 34 protein-coding (*cox2* and *atp9* with two copies), 19 transfer RNA (tRNA), and three ribosomal RNA (rRNA) genes. Phylogenetic analysis showed that *Pedicularis rex* was closely related to *Castilleja paramensis*.

*Pedicularis* L. (Orobanchaceae) is the largest genera of hemiparasitic plants. It has around 600 species, and more than 350 species are restricted into the Himalaya-Hengduan Mountains at the cold and high-latitude habitats (Yang et al. 1998; Yu et al. 2015). In this study, we *de novo* assembled the first complete mitogenome of *Pedicularis rex* C. B. Clarke ex Maximowicz, which is endemic to SW China. The voucher specimen (*W.-B. Yu* et al. *HW10086*, KUN) was collected from Hutiaoxia, Shangri-La, NW Yunnan, China (27°21′00ʺN, 99°54′36ʺE).

Total genomic DNA was extracted from silica gel dried leaves using a modified CTAB method (Doyle and Doyle 1987). Then, the purified DNA was fragmented to ∼500 bp in size for library construction following the Kit protocol (NEBNext® Ultra II™DNA Library Prep Kit for Illumina^®^) (Zeng et al. 2018). The library was performed with 150 bp pair-end reads sequencing using Illumina Hi-seq 2500. Around 6.72 Gb clean data with 39,878,572 reads were used for *de novo* assembling a mitogenome by GetOrganelle toolkit (Jin et al. 2018). To verify the exported complete mitogenome, we checked the assembly graph using Bandage (Wick et al. 2015), showing that organelle contigs sharing between plastome and mitogenome. Read mapping assessing the quality of mitogenome assembly showed that there are 569,559 mitogenome-reads (1.428%) with 387.45 ± 372.58 in the coverage depth and 0.0083 ± 0.0829 in the error rate. The mitogenome was annotated by Ge-seq (Tillich et al. 2017), then manually adjusted using Geneious (Biomatters Ltd, New Zealand).

The mitogenome of *Pedicularis rex* was a single circular chromosome that was 219,859 bp in length (accession no. MN908588). It included 54 unique genes, including 32 protein-coding genes, 19 transfer RNA (tRNA), and three ribosomal RNA (rRNA) genes. The mitogenome GC content was 45.6%. Long repeats, a pair of large repeats 10, 533 bp, and three pairs of small repeats between 100 and 200 bp in length, covered 10.37% (22,786 bp) of the genome in length. The plastid-origin contigs covered 4.49% (9,864 bp) of the mitogenome.

Thirty-two protein-coding genes of 23 species were aligned using MAFFT (Katoh and Standley 2013). Phylogenetic tree was constructed by RAxML (Stamatakis et al. 2008) using GTRGAMMAI model with 1000 bootstrap replicates. Phylogenetic analysis showed that *Pedicularis rex* and *Castilleja paramensis* F. González & Pabón-Mora (Orobanchaceae) formed a clade with 100% bootstrap values, then sister to *Mimulus guttatus* DC (Phrymaceae) (Figure 1). The mitogenome of *P. rex* will provide new insight into evolutionary biology of mitogenome in *Pedicularis*, as well as in Orobanchaceae and other parasitic plants.

**Figure 1.**
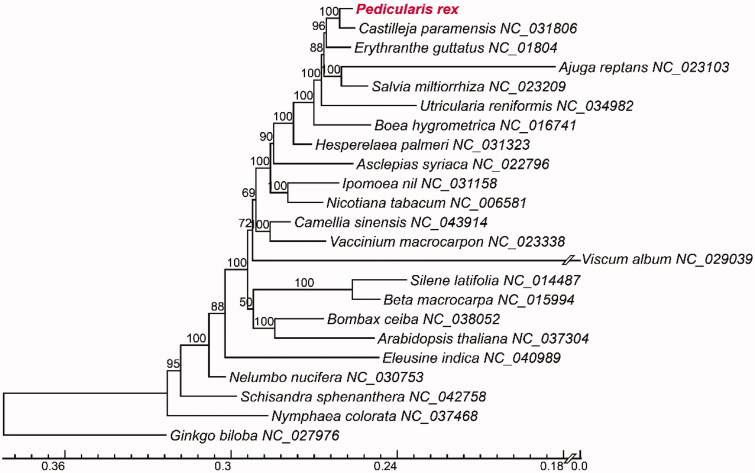
Phylogeny of *Pedicularis rex* and 22 species based on 32 CDS genes using maximum likelihood methods with bootstrap values on the branch. The bottom scale bar represents the number of substitutions per site.
